# Hypomimia in Parkinson's Disease: What Is It Telling Us?

**DOI:** 10.3389/fneur.2020.603582

**Published:** 2021-01-25

**Authors:** Teresa Maycas-Cepeda, Pedro López-Ruiz, Cici Feliz-Feliz, Lidia Gómez-Vicente, Rocío García-Cobos, Rafael Arroyo, Pedro J. García-Ruiz

**Affiliations:** ^1^Department of Neurology, Hospital Universitario Quironsalud Madrid, Madrid, Spain; ^2^Department of Clinical Medicine, Universidad Europea Madrid, Madrid, Spain; ^3^Department of Neurology, Fundación Jimenez Diaz, Madrid, Spain; ^4^Department of Medicine, Universidad Autónoma de Madrid, Madrid, Spain

**Keywords:** facial bradykinesia, Parkinson's disease (PD), hypomimia, amimia, motor symptom, non-motor symptom

## Abstract

**Introduction:** Amimia is one of the most typical features of Parkinson's disease (PD). However, its significance and correlation with motor and nonmotor symptoms is unknown. The aim of this study is to evaluate the association between amimia and motor and nonmotor symptoms, including cognitive status, depression, and quality of life in PD patients. We also tested the blink rate as a potential tool for objectively measuring upper facial bradykinesia.

**Methods:** We prospectively studied amimia in PD patients. Clinical evaluation was performed using the Unified Parkinson's Disease Rating Scale (UPDRS) and timed tests. Cognitive status, depression, and quality of life were assessed using the Parkinson's Disease Cognitive Rating Scale (PD-CRS), the 16-Item Quick Inventory of Depressive Symptomatology (QIDS-SR16), and the PDQ-39, respectively. Amimia was clinically evaluated according to item 19 of UPDRS III. Finally, we studied upper facial amimia by measuring resting blink frequency and blink rate during spontaneous conversation.

**Results:** We included 75 patients. Amimia (item 19 UPDRS III) correlated with motor and total UPDRS (r: 0.529 and 0.551 Spearman), and its rigidity, distal bradykinesia, and motor axial subscores (r: 0.472; r: 0.252, and r: 0.508, respectively); Hoehn and Yahr scale (r: 0.392), timed tests, gait freezing, cognitive status (r: 0.29), and quality of life (r: 0.268) correlated with amimia. Blinking frequency correlated with amimia (measured with item 19 UPDRS), motor and total UPDRS.

**Conclusion:** Amimia correlates with motor (especially axial symptoms) and cognitive situations in PD. Amimia could be a useful global marker of overall disease severity, including cognitive decline.

## Introduction

Hypomimia, amimia, facial bradykinesia, or reduced facial expression (amimia for short) is present in several conditions, including dementia and depression (1–4). Amimia is one of the most classical features of Parkinson's disease (PD) (1, 2, 4–8). It has been well recognized since classic texts (9, 10). In 1860, Charcot described the characteristics of “masked face” in PD (11), and some years later, Wilson gave a vivid description of the parkinsonian facial expression: “The parkinsonian face is a mask,” “the patient has a reptilian stare,” and “little or no play of expression animates his/her countenance.” Wilson used the term *amimia* to include this typical facial signature of PD (10).

Cattaneo et al. summarized some interesting aspects of human facial expression (12). Facial movements differ from limb movements in critical characteristics, including the lack of joints, visual feedback, the conventional proprioceptive feedback system (13), and the absence of the characteristic triphasic EMG pattern seen in limb movements (14, 15). In addition, there is a dissociation between voluntary and emotional facial movements, critically influenced by the amygdala and the limbic system (12).

More recently, Bologna and Marsili summarized the main characteristics and distinctive physiological features of facial bradykinesia in PD (7, 8). Amimia is a peculiar parkinsonian sign: in contrast to limb bradykinesia, amimia is rarely asymmetric (16–18), and it may be present in very early stages of the disease, being evident often years before the clinical diagnosis of PD (19, 20).

Nevertheless, despite being a well-known clinical sign of PD, the relationship between amimia and other motor and nonmotor symptoms of PD is largely unknown.

The main objective of this study is to evaluate the association between amimia and motor and nonmotor symptoms, including cognitive status, depression, and quality of life in PD patients. We also study whether amimia correlates with motor complications, such as freezing of gait and dyskinesias. In addition, we tested the blink rate as a potential tool for objectively measuring upper facial bradykinesia.

## Method

Patients were recruited between December 2016 and June 2018 from the outpatient Movement Disorders Units of Hospital Universitario Quironsalud Madrid and Hospital Fundación Jiménez Díaz (Madrid).

The diagnosis of PD was made according to the UK Brain Bank criteria definition (21). Patients with features consistent with atypical parkinsonism were excluded from the study (such as early and severe loss of postural reflexes, supranuclear gaze abnormalities, dementia during the first 2 years, or significant autonomic symptoms). Patients were also excluded if they suffered from any clinical condition that could potentially affect gait, mobility, or facial movements (including facial paralysis or hemifacial spasm, among others). The clinical evaluation of amimia was based on item 19 of UPDRS III. We also studied upper facial bradykinesia by measuring blinking frequency (the blinking rate was defined as the number of blinks per minute) both resting and during spontaneous conversation. Facial evaluations were recorded on video in order to calculate the blinking rate.

The PD clinical assessment was performed using the Schwab and England Activities of Daily Living Scale, the Hoehn and Yahr Scale, the Unified Parkinson's Disease Rating Scale (UPDRS) (22), and the four timed tests of the CAPIT protocol (23, 24) [including pronation–supination (PS), finger dexterity (FD), movement between 2 points (MTP), and the walking test (WT)]. Tremor score was calculated as the sum of items 20 (tremor at rest) and 21 (action or postural tremor) of UPDRS III; rigidity score was the sum of item 22 of UPDRS III (rigidity of neck and upper and lower extremities); distal bradykinesia score was the sum of items 23 (finger tapping), 24 (hand movements), 25 (rapid alternating movements), and 26 (leg agility); and axial motor score was calculated as the sum of items 18 (speech), 27 (arising from chair), 28 (posture), 29 (gait), and 30 (postural stability) of UPDRS III (25).

Patients were classified according to their main symptom into akinetic-rigid PD, tremor-dominant PD, or mixed PD.

Cognitive and psychiatric aspects of PD were assessed using the Disease-Cognitive Rating Scale (PD-CRS), the 16-Item Quick Inventory of Depressive Symptomatology (QIDS-SR16), and the Parkinson's Disease Questionnaire (PDQ-39).

All patients were evaluated in the mid-morning, after taking their regular medication, in a stable ON condition in order to assess the clinical condition of our patients as similarly as possible to everyday clinical practice.

The present study was performed in accordance with the ethical standards of the WMA Declaration of Helsinki and was approved by the Local Ethics Committee. Written informed consent was obtained from all participants.

### Statistical Analyses

Qualitative variables were expressed as absolute (*n*) and relative (%) frequencies. The Shapiro-Wilk test was used to evaluate the normality of the quantitative variables. Mean values ± standard deviation (SD) are given for normal distributions; for non-normal distributions, the data are reported as medians with interquartile range (IQR).

The correlation between amimia (item 19 UPDRS III) and the continuous variables was performed using the Spearman correlation. For quantitative variables, Student's *t* or Mann-Whitney *U* tests (depending on the normality distribution) were applied to analyze differences between dementia and nondementia group values; chi-square or Fisher's exact test were used for qualitative variables. Finally, univariable linear regression analyses were performed between amimia and age, disease duration, UPDRS and its subscores, rigidity, distal bradykinesia, motor axial, freezing, PD-CRS, PDQ39, and blink rate. Multivariable analyses were performed with the variables that resulted in significant association.

Data analysis was performed with the IBM-SPSS statistical software program, version 21.0 (IBM Inc., Chicago, IL, USA). The significance level was set as *p* < 0.05.

## Results

We included 75 PD patients. Demographic and descriptive results are shown in Table 1.

**Table 1:** Demographic and descriptive results.

	**Total (*n* = 75)**
Age, mean ± SD	70.7 ± 9.6
Sex	
Female, *n* (%)	29 (38.7)
Male, *n* (%)	46 (61.3)
Motor subtype	
Akinetic-dominant, *n* (%)	35 (46.7)
Tremor-dominant, *n* (%)	31 (41.3)
Mixed, *n* (%)	9 (12.0)
LED, median [IQR]	375.0 [520.0]
H and Y, median [IQR]	2.0 [1.5]
SE, median [IQR]	90.0 [20.0]
MDS-UPDRS ON: UPDRS Total, median [IQR]	28.0 [28.0]
MDS-UPDRS ON: UPDRS I, median [IQR]	2.0 [3.0]
MDS-UPDRS ON: UPDRS II, median [IQR]	6.0 [10.0]
MDS-UPDRS ON: UPDRS III, median [IQR]	20.0 [13.0]
UPDRS-19, median [IQR]	2.0 [1.0]
Timed test: PS, median [IQR]	18.6 [28.1]
Timed test: FD, median [IQR]	19.2 [60.9]
Timed test: MTP, median [IQR]	15.7 [28.3]
Blink rate: Resting, median [IQR]	11.5 [17.0]
Blink rate: Conversation, median [IQR]	16.0 [19.0]
PD-CRS: Total, mean ± SD	76.4 ± 24.2
PD-CRS: Cortical, median [IQR]	28.0 [4.0]
PD-CRS: Subcortical, mean ± SD	50.2 ± 21.0
QUIDS-16, median [IQR]	6.0 [7.3]
PD duration, median [IQR]	5 [7]
PDQ-39, median [IQR]	19.2 [27.6]

*PD, Parkinson's Disease; LED:L-dopa equivalent dose; H and Y, Hoehn and Yahr scale; SE, Schwad and England Activities of Daily Living Scale; UPDRS, the Unified Parkinson's Disease Rating Scale; PS, pronation–supination; FD, finger dexterity; MPT, movement between 2 points; WT, walking test; UPDRS 19, Item 19 of the UPDRS III; PD-CRS, Disease Cognitive Rating Scale; QUIDS-16, the 16-Item Quick Inventory of Depressive Symptomatology; PDQ-39, the Parkinson's Disease Questionnaire*.

**Figure 1:**
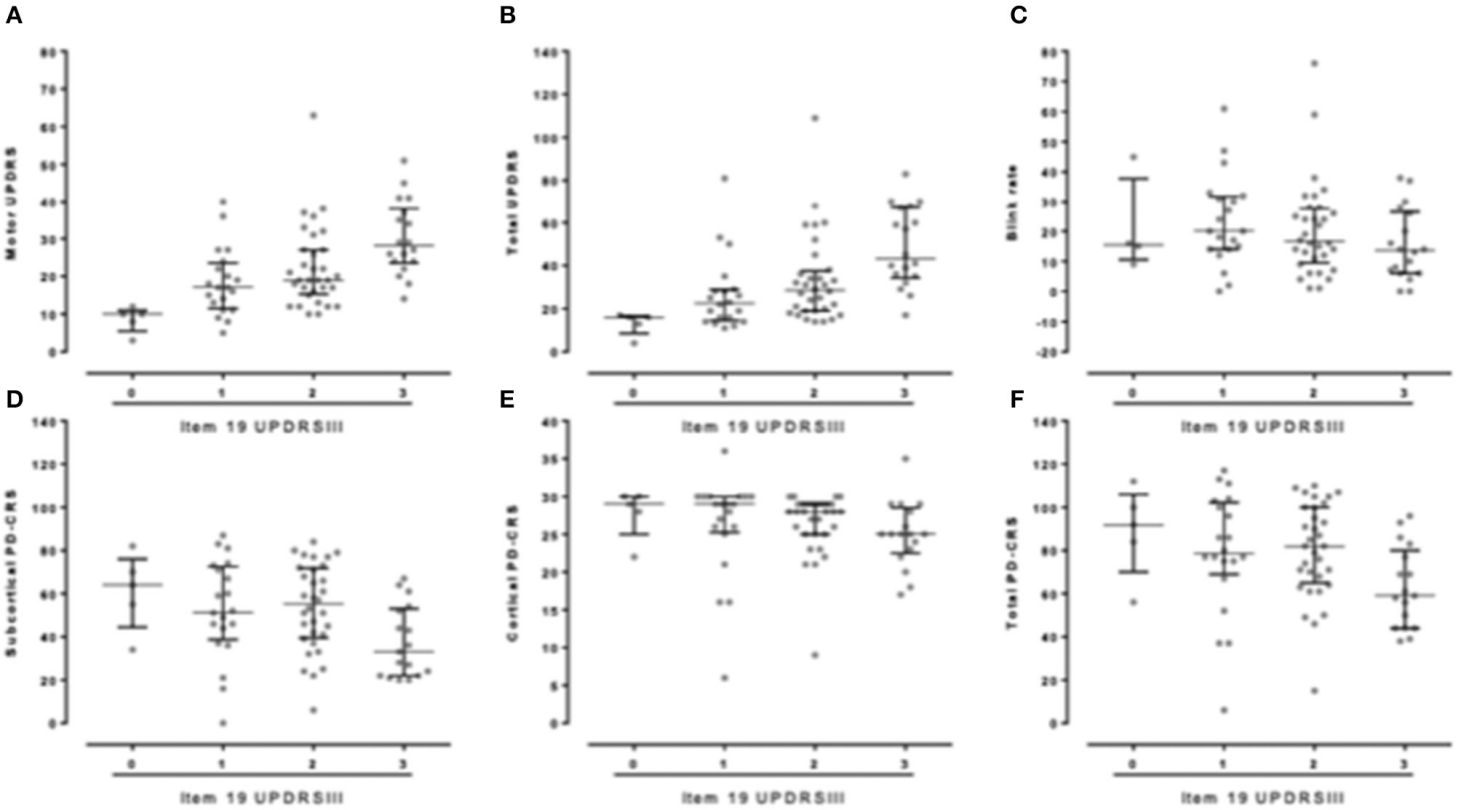
**(A,B)** Correlation between facial bradykinesia (item 19UPDRS III) and UPDRS (**A**. motor-UPDRS; **B**. Total UPDRS). **(C)** Correlation between facial bradykinesia (item 19UPDRS III) and blink rate at rest. **(D–F)** Correlation between facial bradykinesia (item 19UDPRS III) and cognitive test. **(D)** PD-CRS subcortical score; **(E)** PD-CRS cortical score; **(F)** Total PD-CRS.

1) Amimia (measured by item 19 UPDRS III) correlated with clinical scales including total (*p* < 0.01, r: 0.551) and motor UPDRS (*p* < 0.01, r: 0.529) (Figures 1A,B). Amimia correlated with rigidity score (*p* < 0.001, r: 0.472), distal bradykinesia score (*p*: 0.029, r: 0.252), and axial motor score (*p* < 0.001, r: 0.508). In contrast, amimia did not correlate with tremor score.

2) Amimia scores also correlated with bradykinesia measured by timed tests: PS (*p* < 0.05, r: 0.257), FD (*p* < 0.01, r: 0.395), and MTP (*p* < 0.01, r: 0.437) (Table 2).

**Table 2:** Main correlation results using Spearman test.

	**Mimic (Item 19 UPDRS III)**
Total UPDRS	*r* = 0.551; *p* < 0.01
UPDRS III	*r* = 0.529; *p* < 0.01
Rigidity score	*r* = 0.472; *p* < 0.01
Distal bradykinesia score	*r* = 0.252; p = 0.029
Motor axial score	*r* = 0.448; *p* < 0.01
Timed test	
PS	*r* = 0,257; *p* < 0.05
MTP	*r* = 0.437; *p* < 0.01
FD	*r* = 0.395; *p* < 0.01
Freezing episodes	*r* = 0.282; *p* = 0.016
PD- CRS	
Total	*r* = −0.290; *p* = 0.010
Cortical	*r* = −0.270; *p* = 0.019
Subcortical	*r* = −0.290; *p* = 0.011
PDQ-39	*r* = 0.268; *p* = 0.020
Dyskinesia	*r* = −0.391; *p* < 0.01

*UPDRS, the Unified Parkinson's Disease Rating Scale. PS, pronation–supination; FD, finger dexterity; MPT, movement between 2 points; PD-CRS, Disease Cognitive Rating Scale; PDQ-39, the Parkinson's Disease Questionnaire*.

3) Concerning nonmotor symptoms, amimia correlated with cognitive performance, including PD-CRS total, cortical, and subcortical (*p* = 0.010, r:−0.29; *p* = 0.019, r:−0.27; and *p* = 0.011, r:−0.29) (Figures 1D–F). Cognitive decline (defined as PD-CRS ≤ 64) (26) also correlated with amimia (*p* < 0.05; *r* = −0.282). The distribution score for amimia was clearly different between those patients with dementia compared with those without dementia (*p* < 0.05) (Table 3). In contrast, no correlation was found between amimia and depression scores.

**Table 3:** Comparison between patients with and without dementia.

**Item19 UPDRS III**	**Dementia group (*n* = 23)**	**Nondementia group (*n* = 51)**	***P*-value**
Value, mean ± SD	2.2 ± 0.9	1.7 ± 0.8	0.016
Categorical value:			0.044
0	1 (4.3)	4 (7.8)	
1	4 (17.4)	16 (31.4)	
2	8 (34.8)	24 (47.1)	
3	10 (43.5)	7 (13.7)	

*In the first line are results of the Mann-Whitney U test (p: 0.016). The rest of the lines show the values for the chi-squared study for all the values of the UPDRS III item 19 (p < 0.05)*.

4) Regarding motor complications, amimia correlated with the frequency of gait freezing episodes (*p* < 0.05, r: 0.282) and with the presence of dyskinesias (*p* < 0.01, r:−0.391) (Table 2).

5) We also found correlation between amimia and PDQ-39 scores (*p* < 0.05, r: 0.268) and disease duration (*p* < 0.01, r: 0.378) (Table 2).

6) Upper facial bradykinesia measured by resting blink frequency correlated with total UPDRS (*p* < 0.01, r:−0.30), motor UPDRS (*p* < 0.05, r:−0.246), and cortical PD-CRS (*p* < 0.05; r:−0.262) but not with the rest of the studied variables. Amimia measured by item 19 UPDRS III correlated with resting blink rate (*p* < 0.01; r:−0.36) (Figure 1C), but not with blink frequency while participating in spontaneous conversation (*p* > 0.05, r: −0.198).

7) Finally, we carried out a multivariable regression analyses taking into account age, UPDRS III, rigidity, axial and distal bradykinesia subscores, freezing of gait, and cognition. Amimia was the dependent variable. Only distal bradykinesia (ß−0.500; 95% CI−0.178-0.08, *p* < 0.05) and UPDRS III scores (ß 1.155; 95% CI−0.019-0.155, *p* < 0.05) were significant.

## Discussion

The current study was designed to explore the relationship between amimia and other motor and nonmotor symptoms of PD and to evaluate the clinical impression that patients with higher amimia scores have a more severe illness, not only regarding motor symptoms, but also in terms of cognitive status.

Although loss of facial expression is a recognized parkinsonian sign that is well described by classic authors (9–11), its significance and correlation with other PD symptoms is poorly understood. Amimia is one of the most distinctive clinical features in PD and may be one of the earliest symptoms (19). However, in many cases, early amimia might be misinterpreted as lack of interest or depression by attending physicians and families (27). Bologna et al. summarizes several distinctive characteristics of facial expression in PD (7). It is worth recalling that rigidity and tremors scarcely affect the face in PD (15), amimia is rarely asymmetric (7, 16–18), and its response to levodopa and DBS is highly variable (14). For all these reasons, amimia is a peculiar PD symptom, much more laborious to assess than the rest of the PD signs. Furthermore, at this moment, the lack of objective tools to measure amimia makes its evaluation even more difficult.

Our sample shows the usual characteristics of a typical PD population sample (28). In our series, amimia correlated with most motor and nonmotor symptoms. It correlated with motor and total UPDRS scores, and in addition, amimia correlated with timed tests, an objective measure of bradykinesia. Globally, amimia is an indicator of motor impairment as a whole, including axial symptoms and freezing of gait. These results are in line with those from a very recent article published by Ricciardi et al. in which patients with hypomimia had a more severe burden of motor symptoms and higher axial scores (29).

A remarkable result of our study is the possible relationship between amimia and other axial symptoms, such as gait freezing. The results of Riccardi et al. support our findings, describing that patients with amimia have more severe axial symptoms (29). Previous reports also show that patients with freezing of gait often present a nontremor phenotype and have a worse cognitive status (30–32), but to the best of our knowledge, this is the first time that the relationship between amimia and gait freezing has been directly studied.

In our study, amimia also correlated with cognitive status measured by standard scales. Indeed, patients with dementia had greater amimia scores compared with the nondementia PD group. Previous studies report contradictory results about the relationship between amimia and dementia. Recently, Ricciardi et al. find that facial expression is not related to cognitive impairment (29) although Gasca-Salas suggests that *de novo* PD patients with mild cognitive impairment have more severe hypomimia than patients with normal performance in cognition tests (33). The cohort of Riccardi is younger than ours, which could explain, at least in part, the discrepancies in our results (70.7 ± 9.6 in our series vs. 60.3 ± 6.75) (29). According to our findings, the relationship between amimia and cognitive impairment is supported by previous reports in which patients with Lewy Body Disease exhibited more facial bradykinesia compared with PD patients (2, 34–36).

Amimia can be misdiagnosed as depression, a nonmotor symptom of PD (4); however, the relationship between amimia and depression is unclear. Although some studies associate amimia with poor facial emotion recognition and impaired simulated facial expressions (7, 36–38), we find no correlation between depression and facial bradykinesia as previously described (4, 29).

Finally, we aimed to develop an objective method for assessing amimia. In contrast to limb bradykinesia, facial bradykinesia is difficult to estimate based on clinical assessment (14). In order to find a complementary and more objective score than item 19 of UPDRS III, we studied upper facial bradykinesia by measuring both spontaneous resting blink rate and that during conversation. Unfortunately, blink rate correlated poorly with other symptoms of PD. However, resting blink frequency correlated with some motor scores (UPDRS III) as reported by Agostini and Korosec (39, 40), but it had a poor correlation with nonmotor symptoms. Additionally, spontaneous speaking blink frequency was a poor predictor of facial bradykinesia. Although most patients with PD hypomimia have a decrease in blinking frequency, it is suggested that some patients with advanced PD may have an increased spontaneous blink frequency as a form of dystonia (41). Our results suggest that the assessment of amimia should evaluate facial motility as a whole and not upper face motility alone.

Limitations of the present study include the difficulty in making an objective evaluation of amimia as item 19 UPDRS is the only clinically validated tool. At the same time, we did not evaluate the effect of dopa therapy because we assessed patients in the ON situation in order to have a sample as representative as possible of everyday patients. In addition, patients with different levels of severity were included, and for some of them, it would have been difficult to come to our outpatient clinic in an OFF condition.

On the other hand, this study was conducted prospectively on a wide spectrum of idiopathic PD patients with different cognitive and functional situations. We used a comprehensive clinical and cognitive study, including objective timed-test assessment of bradykinesia.

In conclusion, our results suggest that amimia is a potential predictor of global PD severity, including axial symptoms and cognitive decline. Nevertheless, an objective measurement of amimia that is more accurate than UPDRD19 is needed.

## Data Availability Statement

The datasets presented in this study can be found in online repositories. The names of the repository/repositories and accession number(s) can be found at (42).

## Ethics Statement

The studies involving human participants were reviewed and approved by Comite De Ética De La Investigacion De La Fundacion Jimenez Diaz. The patients/participants provided their written informed consent to participate in this study. Written informed consent was obtained from the individual(s) for the publication of any potentially identifiable images or data included in this article.

## Author Contributions

TM-C and PG-R were involved in the study concept and design, the acquisition, analysis, and interpretation of study data, and in the critical revision of the manuscript for important intellectual content. PL-R collaborated in the acquisition of study data and in the critical revision of the manuscript for important intellectual content. CF-F, LG-V, and RG-C worked in the acquisition of data. RA participated in the study design and in the critical revision of the manuscript for important intellectual content. All authors have approved the final version for submission.

## Conflict of Interest

The authors declare that the research was conducted in the absence of any commercial or financial relationships that could be construed as a potential conflict of interest.
